# Genomic amplification of chromosome 7 in the Doxorubicin resistant K562 cell line

**DOI:** 10.6026/97320630014587

**Published:** 2018-12-29

**Authors:** Sara.M Ibrahim, Sajjad Karim, Heba Abusamra, Peter N Pushparaj, Jalaluddin A Khan, Adel M Abuzenadah, Mamdooh A Gari, Sherin Bakhashab, Farid Ahmed, Mohammed H Al-Qahtani

**Affiliations:** 1 Department of Biochemistry, Faculty of Science, King Abdulaziz University, P.O. Box 80218, Jeddah, 21589, Kingdom of Saudi Arabi; 2 Center of Excellence in Genomic Medicine Research, King Abdulaziz University, P.O. Box 80216, Jeddah, 21589, Kingdom of Saudi Arabia; 3 King Fahad Medical Research Center, King Abdulaziz University, P.O. Box 80216, Jeddah, 21589, Kingdom of Saudi Arabia; 4 Faculty of Applied Medical Sciences, King Abdulaziz University, P.O. Box 80216, Jeddah, 21589, Kingdom of Saudi Arabia; 5 KACST Technology Innovation Center in Personalized Medicine, King Abdulaziz University, Jeddah 21589, Saudi Arabia

**Keywords:** Acute myeloid leukemia, multi drug resistance, chromosome 7 amplification

## Abstract

Acquisition of multi-drug resistance (MDR) is a major hindrance towards the successful treatment of cancers. Over expression of a range of ATP-dependent efflux pumps, particularly ABCB1 is a widely reported mechanism of cancer cell MDR. Approximately 30% acute myeloid leukemia (AML) patients demonstrate ABCB1 over expression. Several mechanisms for up regulation of ABCB1 have been proposed. Our aim was to investigate the role of genomic amplification of the chromosome 7 region with regard to its influence on ABCB1 over expression in AML cell line. For this, we developed Doxorubicin (Dox) resistant leukemic cell line from K562 cells, demonstrating MDR phenotype. The chromosomal changes associated with the acquisition of MDR were characterized by array- based comparative genomic hybridization (aCGH) with the parental K562 cell line as the reference genome. Significant genomic gains in the chromosomal region corresponding to 7q11.21-7q22.1 were observed in Dox selected cell line. Moreover, the amplicon contains the ABCB1 gene locus at 7q21.1 with a copy number gain of >4. ABCB1 mRNA was found to be up-regulated by54-fold. Our results demonstrate that the development of MDR in K562/Dox is underlined by a genomic amplification of the chromosome 7 region harboring the ABCB1 gene.

## Background

Acute myeloid leukemia (AML) is a complex and heterogeneous disease with poor clinical outcome, especially in the elderly who constitute the majority of AML patients 1. The treatment strategies for selected subtypes of AML has recently started to change 2. However, for the majority of the AML patients, conventional chemotherapy remains the frontline therapeutic choice. In younger AML patients, 70-80% achieve complete remission (CR) but most of them eventually relapse and overall survival (OS) in this group is only 40-50% at 5 years 3. The OS in older patients is worse with a cure rate of less than 10%. The primary reason for the failure of AML therapy is drug resistance. Multidrug resistance (MDR), which is the simultaneous acquisition of resistance to different chemotherapeutic agents, is a daunting clinical challenge in AML. Understanding the underlying mechanisms of MDR is therefore essential to improve the efficacy of chemotherapeutic agents as well as to design newer agents to overcome MDR.

The study of resistant cell lines in-vitro has contributed to the identification and characterization of important mediators of cancer cell MDR. MDR phenotype in cultured cells was characterized by amplification of the genomic segment containing the ATP binding cassette sub family B member1 (ABCB1) gene 4. ABCB1 gene, cytogenetically mapping to 7q21.12, encodes the ABCB1 efflux pump that plays a critical role in the active uptake and transport of several molecules, including anticancer therapeutics across the cell membrane. ABCB1 over expression is a widely reported mechanism of MDR 5. In AML, increased expression of ABCB1 is associated with therapy resistance and poor prognosis 6.

Human chromosome 7 is approximately 159Mb in length and contains 1150 genes and 940 pseudogenes. Aberrations in chromosome 7 are implicated in the pathogenesis of several diseases such as cystic fibrosis, deafness, autism, andcancer. Several cancer models have reported genetic changes and unstable regions in chromosome 7. A number of oncogenes including epidermal growth factor receptor (EGFR), hepatocyte growth factor receptor and MET proto-oncogene (c MET) and v-raf murine sarcoma viral oncogene homolog B1B-Raf proto-oncogene, serine/threonine kinase (BRAF) are located on chromosome 7 7. Thus, chromosome 7 copy number changes i.e. aneuploidy/polysomy/monosomy or gene abnormalities due to mutation/amplification plays a crucial role in different malignancies including solid tumors, lung, colon, and head and neck carcinomas. Changes in chromosomal 7 regions were also reported in gastric cancer disease modeling and progression 8. The study characterized the genes undergoing significant copy number variations in gastric cancer tissue, as protooncogenes and genes involved in signal transduction pathways regulating proliferation, metabolism, transport, inflammatory response, and proteolysis. Furthermore, several studies have reported amplification of the chromosome 7q region containing ABCB1 gene in MDR 9-14.

In AML, chromosome 7 aberration, specifically monosomy and deletion of the long arm, is singularly observed in approximately 4- 5% newly diagnosed AML patients, the incidence being higher as a part of complex karyotypic AML feature 15. Patients with chromosome 7 deletion in the absence of other abnormalities are considered under intermediate risk group while chromosome 7 monosomy, either alone or combined with other chromosomal abnormalities, present a more adverse prognosis 16. A study by Slovak et al showed the clinical outcome of patients with complex karyotype containing abnormalities of chromosomes 7 i.e. deletion/monosomy was poor and associated with lower CR and survival rates 17. Another study reported drug resistance and early death in 77.8% AML patients with the associated chromosomal 7 aberrations 18. Abnormalities in chromosome 7 are also frequently encountered with therapy related AML, particularly with the use of alkylating agents 19. This is further evident by several studies wherein deletion affecting the long arm of chromosome 7 was recurrently acquired at relapse 20,21 and presented as a marker of poor prognosis at diagnosis, thereby underlining its significance in therapy resistance and refractory AML. The exact pathogenic role of chromosome 7 monosomy/deletion in leukemogenesis is, however, unclear and is largely hypothesized to be related to loss of a critical tumor suppressor gene 22. Association between chromosome 7 abnormalities and ABCB1 over expression was also reported 23.

There are a limited number of studies mapping the chromosomal changes contributing to disease relapse in AML. An insight into the associated chromosomal alterations is hence, integral in understanding mechanisms of acquired resistance and potential targets to overcome MDR. Comparative genomic hybridization (CGH) enables the detection and localization of genome-wide chromosomal aberrations i.e. amplifications and deletions of a test sample relative to a reference. Genomic DNA from the resistant and parental cell line was comparatively hybridized that enabled the identification of genetic aberrations contributing to drug resistance. In the present study, we have focused on chromosome 7 amplification in the development of MDR resistance in Doxorubicin (Dox) selected AML cell line.

## Methodology

Doxorubicin, daunorubicin, idarubicin, etoposide, and tariquidar were purchased from Selleckchem,USA. Verapamil (Merck, V4629) was provided as a kind gift from Dr. Gauthman (CEGMR). QIAamp DNA Mini Kit and RNAeasy Mini Kit were purchased from Qiagen, USA. SuperScript VILO cDNA Synthesis Kit and Power SYBR Green PCR Master Mix were purchased from ThermoFisher Scientific, USA.

### Cell culture:

K562 cells (CLS GmBH, Germany) were cultured in RPMI media (Gibco, life technologies, USA) supplemented with 10% FBS at 37°C in a humidified incubator K562/Doxcell line was obtained by culturing K562 cells in gradually increasing dose of Dox (10nM- 200nM). Cells were grown in drug-free media for at least two weeks before experimentation.

### Cell viability assay:

Cell viability was determined using the CellTitre-Blue Cell Viability Assay from Promega as per manufacturer's protocol. Briefly, 10,000 cells were seeded in 96 well plate and different dilutions of the drugs were added. Plates were incubated for 48h at 37°C. 20µL of the CellTitre-Blue reagent was added and incubated for additional 2h. Fluorescence was measured at 540Ex/590Em on SpectraMax i3x Multi-Mode plate reader (Molecular Devices, USA). The mean inhibitory concentration of the drug (IC50) was obtained using the non-linear regression model.

### Array based comparative genomic hybridization (aCGH):

The aCGH analysis was performed as per manufacturer's protocol using Agilent SurePrint G3 Human CGH 2x400K arrays, Agilent labeling kit (Agilent Technologies, USA). Briefly, 500ng of the reference and sample DNA were digested at 37°C by RsaI and AluI (Promega, USA) for 2h. Sample and reference DNA were labeled with Cy3-dUTP (Green) and Cy5-dUTP (Red) respectively. Labeled samples were purified and size selected using Microcon YM-30 filter units (Millipore, Billerica, Massachusetts, USA). Cot-1 DNA (Invitrogen, Carlsbad, California, USA), hybridization buffer and blocking agent were mixed with labeled DNA and denaturation was performed at 95�C for 5min and maintained at 37°C, before hybridization to the array at 65°C for 40 ± 2h at 20rpm. Microarray slides and gaskets were disassembled and washed. Slides were briefly rinsed with anhydrous acetonitrile followed by a final wash with stabilizing and drying solution. Chip scanning, image analysis, and data extraction were performed on an Agilent Scanner (G2505C), and Agilent's Feature Extraction software (V.1.5.1.0) respectively. Agilent CytoGenomics v2.7 software was used to visualize, detect and analyze aberrations.

### Quantitative real time polymerase chain reaction (qRT-PCR):

Primers were designed using the NCBI database (http://www.ncbi.nlm.nih.gov/nucleotide) and were supplied by Metabion (Steinkirchen, Germany). The Step One Plus Real-Time PCR system (Life Technologies, Paisley, UK) was used for quantification of gene transcripts. The amplifications were performed under the following conditions: 95°C for 20s, followed by 40 cycles of 95°C for 15s, 60°C for 60s and 72°C for 15s. Data were collected at the end of the extension step (72°C). Comparative threshold cycle (ΔΔCt) method was used to quantify the relative expression of the target genes.

### Statistical analysis:

All statistical analysis was performed using GraphPad Prism Software version 6.07 (GraphPad Software, La Jolla, CA, USA). Student's t-test was used to compare paired data points between each group. A p-value less than 0.05 over 95% CI was considered statistically significant.

## Results

### Dox sensitivity in K562 and Dox resistant K562 cell line:

**Figure 1:**
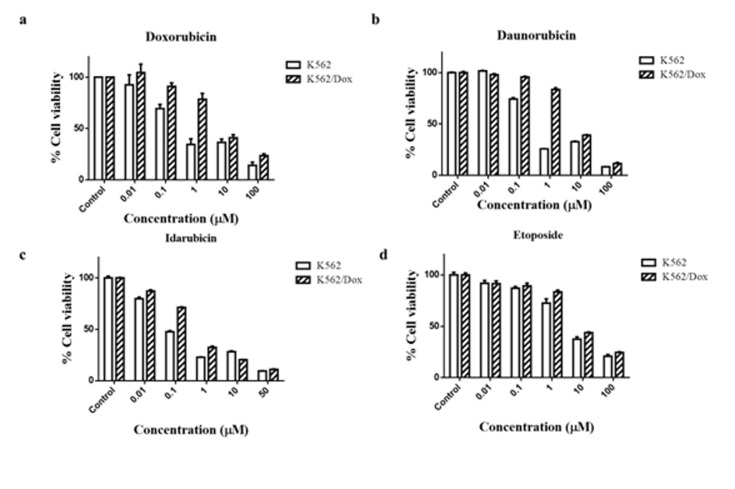
K562/Dox cell line show resistance to different chemotherapeutics. K562 and K562/Dox were incubated with increasing concentrations (0.01-100 micro molar) of a) Doxorubicin, b) Daunorubicin,c) Idarubicin, and d) Etoposide for 48h. IC_50_ was determined by nonlinear regression of data points. Data represents average of three independent experiments performed in triplicates.

Dox-resistant cell lineK562/Dox was derived from K562 parental cells by culturing the cells in gradually incremental doses of Dox (10nM -200nM) followed by clonal selection. Dox cytotoxicity in the parental and resistant cells was determined by CellTiter-Blue cell viability assay. As shown in Figure 1a, the IC50, which is the mean inhibitory concentration producing 50% cell death, for K562/Dox was 3.06 ± 0.266µM as compared to the Dox-sensitive parental K562 cell line, which showed a relatively low IC50 of 0.117 ± 0.003µM. Thus, Dox resistant cell line K562/Dox demonstrating 26-fold higher resistance to the parental K562 cells was obtained. The cytotoxicities of other anthracyclines - daunorubicin and idarubicin werealso assessed in K562 and K562/Dox cell lines.

As shown in Figure 1b and 1c, K562/Dox showed varying levels of cross-resistance to daunorubicin and idarubicin i.e. 6.8-fold and 1.44 -fold respectively when compared to K562 cells. We next tested the cross-resistance to a structurally unrelated, non-anthracycline drug: etoposide, which is a topoisomerase II inhibitor. K562/Dox demonstrated significant increase in etoposide IC50 values from 2.4 ± 0.071µM in K562 to 5.247 ± 0.077µM in K562/Dox (Figure 1d). Since the resistance was not limited to Dox alone and the resistant cell line displayed significantly higher levels of resistance to structurally related and unrelated drugs and to drugs it was not primarily exposed to, it was established that K562/Dox is an MDR cell line. Addition of 5µM verapamil, a calcium ion channel blocker, and a known ABCB1 modifier, decreased K562/Dox fold resistance to 14-fold while complete restoration of Dox sensitivity was observed in K562/Dox upon treatment with 100nM tariquidar, which is a highly potent and specific inhibitor of ABCB1 ( Table 1). Moreover, K562 and K562/Dox did not show a significant difference in the IC50 of cisplatin, an ABCG2 substrate (data not shown). These observations indicate an underlying role of ABCB1 efflux transporter in contributing to the MDR of K562/Dox.

### ABCB1 genomic amplification was observed in Dox-resistant cells:

**Figure 2:**
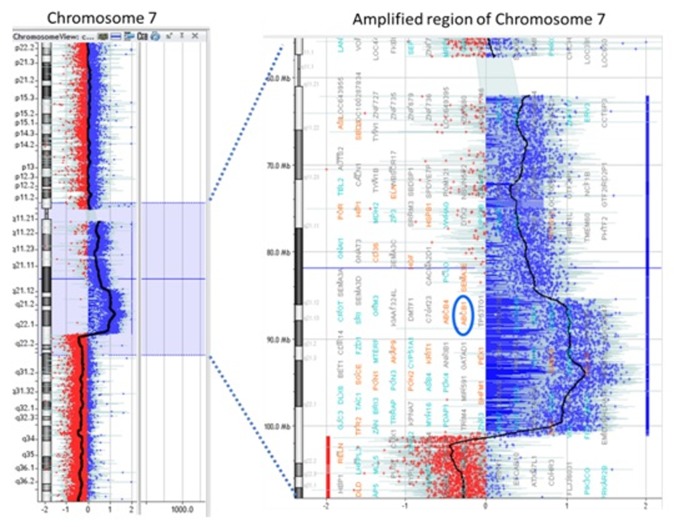
aCGH was performed for K562/Dox using K562 as the reference genome.Graphical view representing amplification in chromosomal 7 region showing gain of ABCB1 gene locus corresponding to 7q21.12 in K562/Dox as compared to K562 cells.

In order to characterize the genomic aberrations associated with the development of MDR whole-genome aCGH was performed for K562/Dox, using parental K562 as the reference genome. Multiple minor genomic aberrations i.e. both gains and losses were observed in chromosome 1, 4, 5, 7, 12 and 16 in K562/Dox, as compared to K562. A major aberration was observed in chromosome 7. The copy number profile revealed gain in the q11.21~q22.1 region of chromosome7, with the amplicon spanning around 39Mb. In particular, high-level amplification was observed for q21.1~q22.1 region displaying >4 copy number gains. The most prominent gene in this region was ABCB1 mapping to the 7q21.12 locus (Figure 2). This was most relevant to our previous findings, explaining the relative resistance of K562/Dox to Dox, daunorubicin, idarubicin, and etoposide, as all the chemotherapeutic drugs are well known substrates of the ABCB1 protein. Thus, ABCB1 copy number gain was recognized as the molecular basis of acquired chemoresistance demonstrated by K562/Dox cell line.

### Dox-resistant cell line overexpress ABCB1 gene:

**Figure 3:**
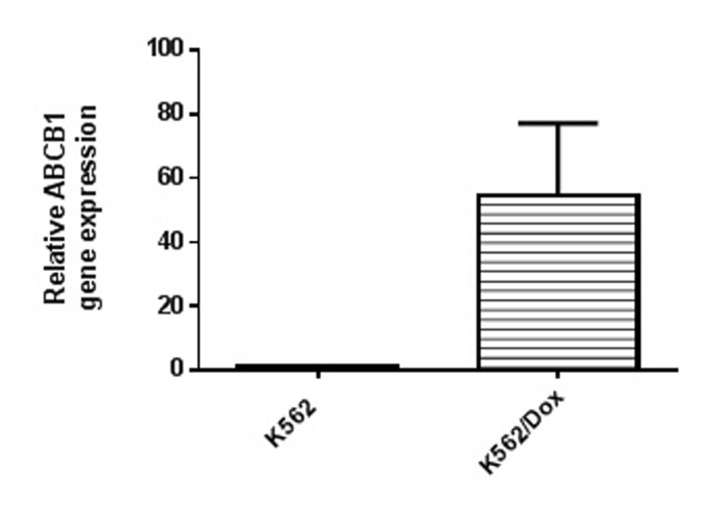
Relative ABCB1 gene expression in K562 and K562/Dox cell lines.

In order to evaluate the increase in ABCB1 gene expression,qRTPCR was performed using the parental K562 as control and GAPDH as the reference gene. ABCB1 mRNA levels were quantified in the parental and resistant cell lines - K562 and K562/Dox. Relative gene expression was calculated by ΔΔCt method. As shown in Figure 3, K562/Dox overexpressed ABCB1 mRNA by 54-fold when normalized to the parental K562 cells. Thus, ABCB1 gene overexpression was confirmed as the predominant mechanism of acquisition of resistance.

## Discussion

ABCB1 over expression is a widely reported mechanism of cancer cell MDR, occurring in approximately 30% of AML patients 1[6]. About 70% of secondary AML patients also show up regulation in the ABCB1 gene expression 24. Cells exposed to ABCB1 substrate chemotherapeutics frequently demonstrate enhanced ABCB1 expression via multiple mechanisms. Our aim was to investigate the role of chromosome 7amplification with regard to its influence on ABCB1 over expression and consequent development of MDR phenotypein AML in-vitro. K562/Dox cell lines derived by culturing K562 cells in gradually increasing doses of Dox displayed varying levels of cross resistance to ABCB1 substrates i.e. daunorubicin, idarubicin, and etoposide and not to cisplatin, an ABCG2 substrate. Moreover, the addition of known ABCB1 inhibitors such as verapamil and tariquidar restored Dox sensitivity in K562/Dox cells. These observations strongly indicate the role of ABCB1 in mediating the observed MDR of K562/Dox.

Genomic amplification of chromosome 7 harboring the ABCB1 gene has been reported in MDR in different types of cancers including lung 12, breast 11, liver 14, neuroblastoma 9, esophageal 10 and ovarian cancers 13. ABCB1 genomic locusalterations have been previously reported in AML cell lines that involved translocation of MDR1 gene to chromosome 2 and subsequent amplification under cytotoxic stress. The removal of the ABCB1 gene from its original chromosomal site was considered an important early event that released the normal regulatory control of chromosome 7 facilitating the observed amplification 25. Increase in copy number of the 7q21~q22 region has also been reported for vincristine selected leukemic cell line 26. In the present study, both low-level gains and high-level amplification were observed in the chromosome 7q region of K562/Dox by aCGH analysis. Particularly, the genomic region containing the ABCB1 gene demonstrated high copy number gain of >4. This was consistent with the ABCB1 expression at the RNA level and the results in cell viability experiments wherein K562/Adr showed co-resistance to ABCB1 substrate chemotherapeutics.

Genomic profiling of the ABCB1 amplicon enables the elucidation of the role of nearby genes in the development of MDR. In K562/Dox cell line, the amplicon spanning around 39Mb comprised of several genes in addition to the ABCB1 gene. Further exploration of additional genes within the amplicon that are conferring or contributing to the MDR phenotype is justified. Some of the genes in the amplicon that have been previously described in MDR include ABCB4, Carnitine O-octanoyl transferase (CROT) and TP53TG1 13,27. ABCB4 is a member of ABC transporter sharing 82% nucleotide sequence homology to the ABCB1 protein 28. ABCB4is often co-amplified with ABCB1 because of their close genomic proximity and is particularly selective for paclitaxel and vinblastine resistance 29. However, the exact role of ABCB4 transporter in MDR is still unclear, as silencing of ABCB4 did not restore sensitivity whereas silencing of ABCB1 completely restored sensitivity in taxane-resistant ovarian cancer cell line. Similarly, CROThas been shown to be co-amplified with ABCB1 in taxaneresistant ovarian and breast cancers 13. TP53TG1 encodes p53- induced lncRNA, activated upon DNA damage and its up regulation has been reported in T-lymphocytes after exposure to ionizing radiation, cisplatin-resistant colon cancer and docetaxelresistant breast cancer 30. The TP53TG1 lncRNA binds to YBX1 protein and prevents the activation of oncogenes. Although cellular lncRNA may increase under cytotoxic stress, it is assumed that epigenetic silencing of the promoter leads to MDR 31. On the other hand, TP53TG1 is shown to promote cell proliferation and migration in glioma cells under glucose deprivation 32.

Thus, co-activation of proximal genes may not be a chance consequence of genomic amplification and it is probable that the amplified genes may have an important independent or concerted role in inducing MDR. This is further evident by the study by Wang et al. that reported activation of a cluster of 22 genes in the 7q21.11- 13 chromosomal region in several taxane-selected ovarian cancer cell lines which was not essentially the result of copy number alterations 13.

Gene expression is regulated by different mechanisms such as genomic amplification, enhanced transcription, mRNA stabilization, post-transcriptional regulation and epigenetic modifications. In the present study, K562/Dox showed remarkably high ABCB1 gene expression as compared to the parental cell line. This was consistent to the observations in paclitaxel resistant lung cancer cell line, wherein ABCB1 gene expression increased upto 1000-fold, while a copy number gain of only 12 was found in the ABCB1 genomic region 12. Thus, it is evident that apart from chromosomal copy number changes, other mechanisms such as chromatin remodeling and DNA or histone modifications of the 7q21 region may contribute to ABCB1 genomic amplification.

## Conclusion

Our data highlight that the genomic amplification of the chromosome 7 region containing ABCB1 results in the up regulation of ABCB1 and hence MDR in K562 human erythro leukemia cells. Involvement of additional genes amplified in the amplicon in the context of drug resistance needs to be investigated.

## Figures and Tables

**Table 1:** Dox sensitivity in K562 and K562/Dox resistant cell line upon indicated treatment.

Treatment	K562 IC50 (µM) ± SD (FR)	K562/Dox IC50 (µM) ± SD (FR)
Doxorubicin	0.117 ± 0.003 (1.00)	3.065 ± 0.266 (26.19)
+5�M Verapamil	0.109 ± 0.053 (0.93)	1.683 ± 0.21 (14.38) **
+100nM Tariquidar	0.116 ± 0.002 (1.06)	0.175 ± 0.001 (1.50) **

**Table 2:** Primer pair sequence used for qRT-PCR

Gene	Amplicon size	Forward primer	Reverse primer	Tm°C
GAPDH	81	GCCATCAATG	GCCATGGAA	58
		ACCCCTTCAT	TTTGCCAT	50
ABCB1	105	TTGCTGCTTAC	AGCCTATCTC	60.7
		ATTCAGGTTTCA	CTGTCGCATTA	60.2
